# Isolation and screening of biosurfactants producing lactic acid bacteria strain from Bhatabharu, an Indian traditional fermented food

**DOI:** 10.5114/bta/207713

**Published:** 2025-08-26

**Authors:** Tamanna Kaundal, Anjali Sharma, Navneet Batra

**Affiliations:** 1 Department of Biotechnology, Panjab University Research Center, Goswami Ganesh Dutta Sanatan Dharma College (GGDSD) College, Chandigarh, India; 2 Department of Biotechnology, DAV College, Chandigarh, India

**Keywords:** biosurfactants, *Enterococcus faecalis*, Indian fermented food, lactic acid bacteria (LAB)

## Abstract

**Background:**

Biosurfactants derived from lactic acid bacteria (LAB) produce eco-friendly biosurfactants with antimicrobial, antiadhesive, and antibiofilm properties.

**Materials and methods:**

LAB strains isolated from Bhatabaru were screened for biosurfactant production using multiple assays, including drop collapse, hemolytic activity, oil displacement, surface activity, and emulsifying activity. The selected strain was morphologically characterized by Gram staining and microscopy and identified through biochemical assays and 16S rRNA sequencing using Gene Tool software.

**Results:**

The strain Bht-2 was determined to be Gram-positive, coccus-shaped, and nonendospore-forming. Biochemical and molecular analyses confirmed its identity as *Enterococcus faecalis*, which exhibited significant biosurfactant production.

**Conclusions:**

*Enterococcus faecalis* Bht-2 exhibits strong potential as a biosurfactant-producing LAB strain. Its desirable physicochemical and biofunctional traits underscore its applicability in biotechnological, pharmaceutical, and industrial domains as a safe and eco-friendly alternative to synthetic surfactants.

## Introduction

India has a long history of fermented food consumption in various forms, primarily prepared from cereals, fruits, fish, dairy, meat, and nuts, influenced by its diverse geographical and climatic conditions. These foods are not only fermented but also functional, offering additional benefits such as enhanced flavor, improved digestibility, and enriched nutritional and pharmacological qualities, alongside extended preservation (Sarma and Gupta 2022). Fermentation by microorganisms induces several changes in food, including the enhancement of dietary protein quality and water-soluble vitamin content.

In the Indian state of Himachal Pradesh, traditional fermented foods such as Seera, Bhaturu, Bari, Jhol, Bhatabaru, and Chhang are consumed as part of the regular diet. Lactic acid bacteria (LAB), including *Lactobacillus, Lactococcus, Enterococcus, Streptococcus, Pediococcus, Leuconostoc*, and *Weissella*, are abundantly present in these fermented foods (Mathur et al. 2020). LABs produce several metabolites such as bacteriocins, biosurfactants, organic acids, and other inhibitory compounds that suppress the growth of harmful microbes (Tang et al. 2023).

Biosurfactants are surface-active molecules produced by microorganisms on their cell surface. These molecules exhibit antiadhesive, antimicrobial, and antibiofilm properties and have been derived from various microorganisms present in fermented foods. Biosurfactants include low molecular weight microbial compounds, such as lipopeptides and glycolipids, which reduce surface and interfacial tension, and high molecular weight compounds, such as polysaccharides, lipopolysaccharides, proteins, or lipoproteins, which help stabilize emulsions (Kumar et al. 2021). Numerous microbes are known to produce biosurfactants, including *Acinetobacter* spp., *Bacillus* spp., *Candida antarctica*, and *Pseudomonas aeruginosa* (Eras-Muńoz et al. 2022).

Among these, *Bacillus* and *Pseudomonas* are known for high-yield biosurfactant production. However, due to the pathogenic nature of some producing microorganisms, their applications are limited. Consequently, nonpathogenic LAB are being explored as potential biosurfactant producers. Compared to synthetic surfactants, LAB-derived biosurfactants are less toxic, biodegradable, and remain active under severe temperature and pH levels (Johnson, 2021). Hence, there is significant potential for using biosurfactants in sectors such as agriculture, bioremediation, cosmetics, and pharmaceuticals. Moreover, synthetic surfactants, due to their persistent nature, pose environmental and toxicological risks, which can be mitigated by the biocompatible and biodegradable nature of biosurfactants (Bjerk et al. 2021).

Given the unique properties of biosurfactants, they serve as a promising alternative to synthetic surfactants. As fermented foods are rich sources of LAB, the present study aimed to explore the biosurfactant-producing potential of LAB isolated from Bhatabaru, a traditional dish from Himachal Pradesh. This cereal-based food is prepared using wheat flour, water, milk, and sugar. Often enjoyed during festivals and special occasions, Bhatabaru reflects the region’s vibrant culture and culinary traditions (Thakur et al. 2004; Tamang et al. 2009).

## Materials and Methods

### Sample preparation

For the isolation of LAB from Bhatabaru, 5 g of wheat (*Triticum aestivum*) flour was placed in a sterile flask, and 25 ml of a water–milk mixture (2 : 1) was added. The mixture was allowed to ferment naturally for 2 h at 37°C under laboratory conditions (Kanwar 2014).

### Isolation of LAB

After fermentation, 0.5 ml of the Bhatabaru slurry was inoculated into 100 ml of sterile MRS (Man-Rogosa-Sharpe agar) broth and incubated at 37°C for 24 h. A 1 ml aliquot of the broth was then serially diluted, and 50 μl of the 10^–6^ to 10^–7^ dilutions was spread onto sterile MRS agar plates. The plates were incubated for 24 h at 37°C under anaerobic conditions. Various colonies that grew on MRS media were streaked multiple times to obtain pure colonies. Only catalase- and oxidase-negative strains were selected for further screening (Jo et al. 2021).

### Selection of biosurfactant-producing isolates by different tests

#### Hemolysis test

The screening of biosurfactant-producing LAB was performed by streaking different isolates on sheep blood agar plates (Himedia MP1301-50PT), followed by incubation at 37°C for 48 h. The plates were observed for hemolytic activity, indicated by the formation of clear lytic zones around the bacterial colonies (Mamta et al. 2020).

#### Oil displacement method

A total of 25 ml of distilled water was added to an empty Petri plate. Then, 20 μl of motor oil was dropped onto the water surface to form an oil layer. Subsequently, 20 μl of cell-free broth from different isolates was added to the oil layer, and the plate was observed for clear zone formation. The presence of biosurfactants in the supernatant leads to oil displacement, which is proportional to the diameter of the clear zone formed on the oil surface (Phulpoto et al. 2020).

#### Emulsifying activity

To measure emulsification activity, 2 ml of cell-free supernatant and 2 ml of vegetable oil were mixed in a test tube. The mixture was vortexed at high speed for 2 min and then left undisturbed at room temperature for 24 h. The emulsification index (E24) was calculated using the following equation (Nayarisseri et al. 2018):

$$\text{Emulsification index}=\frac{\text{Height of the emulsified layer}}{\text{Total height of the column}}\times 100$$

#### Drop collapse method

The drop collapse test was performed by pipetting 10 μl of cell-free broth from different isolates onto parafilm. The droplets were observed for spreading or flattening on the parafilm surface. SDS was used as a positive control, and distilled water served as a negative control. The presence of surfactants in the cell-free supernatant reduces the interfacial tension between the liquid drop and the hydrophobic surface, causing the drop to spread or collapse (Zargar et al. 2022).

### Determination of surface tension

A stalagmometer was used to evaluate the surface tension of the cell-free supernatant using the drop weight method. Distilled water was drawn into the capillary tube up to mark A and then allowed to fall naturally by gravity into a dry beaker. The weight of the droplets was measured using an electronic balance after collecting 30 drops. The same procedure was followed for the cell-free supernatants of various isolates. Modified MRS broth (without Tween 80) was used as a control. The surface tension of each sample was calculated using the following equation (Wei et al. 2005):

$${\text{V}}_{1}=\frac{{\text{W}}_{1}}{{\text{W}}_{2}}{\text{V}}_{2}$$

where *V*_1_ = surface tension of test liquid, *V*_2_ = surface tension of distilled water, *W*_1_ = weight of test liquid, and *W*_2_ = weight of distilled water.

### Biosurfactant production

To produce biosurfactants from the selected strain, 2 ml of an overnight culture was added to 150 ml of sterile modified MRS broth (without Tween 80) and incubated at 37°C for 48 h under static conditions.

### Biomass determination

To determine biomass, 20 ml of inoculated broth was centrifuged in pre-weighed centrifuge vials at 7000 rpm for 20 min. The resulting cell pellet was washed twice with PBS and dried at 80°C in a hot air oven for 24 h. The biomass was then weighed (Lira et al. 2020).

### Biosurfactant extraction and yield determination

After incubation, the culture was centrifuged at 10,000 rpm for 20 min to obtain the cell-free supernatant (CFS). As biosurfactants are extracellularly produced and often adhere to the microbial cell surface, the cell pellet was resuspended in PBS (pH 7.4) to release the adhered biosurfactants. This suspension was then centrifuged at 9000 rpm for 10 min, and the resulting supernatant containing the released biosurfactants was collected.

The CFS was acidified to pH 2 using 2 N HCl to precipitate the biosurfactants and stored overnight at 4°C. Subsequently, three sequential extractions were performed using a chloroform/methanol mixture (2 : 1, v/v), and the organic phase was collected and subjected to vacuum evaporation to remove the solvents. The biosurfactants were then dissolved in acetone. After evaporation, the resulting precipitate was transferred to a pre-weighed sterile glass Petri dish and dried in a hot air oven at 80°C for 30 min to ensure complete solvent removal. The dish was reweighed to determine the biosurfactant yield using the following formula:

$$\text{Yield of biosurfactants}=\frac{\left(\text{Weight of the plate after drying}-\text{weight of the empty plate}\right)}{\left(\text{Biomass concentration}\right)}$$

### Genotypic and phylogenetic characterization of selected LAB

The strain exhibiting superior biosurfactant activity and higher yield was selected among all biosurfactantproducing isolates and identified based on morphological, genetic, and phylogenetic analyses.

### Morphological, biochemical, and genotypical identification of selected strain

The isolated strains were identified by morphological, biochemical, and genetic characterization. LAB identification was initially based on morphological and biochemical tests. Gram staining, negative staining, spore formation, and motility tests were conducted for morphological characterization. Biochemical identification involved tests for indole production, methyl red, Voges-Proskauer reaction, citrate utilization, nitrate reduction, H_2_S production, catalase, oxidase, and carbohydrate utilization (Adikari et al. 2021).

### DNA isolation

After overnight incubation, 4 ml of the culture was centrifuged, and the resulting pellet was washed with PBS before being suspended in 1 ml of lysis solution containing 5 mg of lysozyme and 30 U/ml of mutanolysin. The mixture was incubated at 37°C for 30 min, followed by incubation at 60°C for 50 min. Subsequently, 20 μl of proteinase K (20 mg/ml) and 50 μl of 10% sodium dodecyl sulfate were added.

DNA was extracted five times with phenol–chloroform–isoamyl alcohol (25 : 24 : 1) and twice with chloroform–isoamyl alcohol (24 : 1). DNA was precipitated by adding 0.1 volume of 3 M sodium acetate and two volumes of 96% ethanol at –20°C. The DNA pellet was dissolved in 150 μl of TE buffer (10 mM Tris [pH 8], 1 mM EDTA) containing 20 μg of RNase. DNA concentration was measured using a spectrophotometer at 260 nm absorbance (Abbasiliasi 2012).

### Amplification of DNA, 16S rRNA sequencing, and phylogenetic analysis

The DNA of the selected strain was amplified using 16s F243, 5′-GGATGAGCCCGCGGCCTA-3′ and reverse, R1378 5′-CGGTGTGTACAAGGCCCGGGAACG-3′ primers.

PCR amplification was performed in a 100 μl reaction volume containing 10 μl of 10× PCR buffer, 2 μl of 10 mM dNTPs, 3 μl of 1.5 mM MgCl_2_, 3 μl of 20 pM of each primer, 1 μl of template DNA, 0.7 μl of Taq DNA polymerase (5 U/μl), and 77.3 μl of sterile distilled water. The PCR was carried out using a 30-cycle protocol as described in Table 1.

**Table 1:** Cycling conditions for PCR

Step	Duration	
Initial denaturation	3 minutes at 94°C	30 cycles
Denaturation	1 minute at 94°C	
Annealing	1 minute at 65°C	
Extension	2 minutes at 72°C	
Final extension	7 minutes at 72°C	

The amplified product was separated on 2% agarose gels in 1× TAE buffer for 90 min at 10 V/mm, visualized using a UV transilluminator, and documented using GelDoc XR software (Bio-Rad). The amplification product was purified and submitted to Bioserve Biotechnologies (India) Pvt. Ltd., Hyderabad, India, for sequencing.

Using GeneTool software, the sequences obtained from forward and reverse primers were assembled to generate a consensus 16S rRNA sequence. A BLAST analysis was performed to compare this consensus sequence with those in the NCBI GenBank database. Based on the maximum identity scores, the top ten closely related sequences were selected, and a phylogenetic tree was constructed (Nayarisseri et al. 2018; Khedkar and Shanker 2014).

## Results and Discussion

### Preliminary screening of biosurfactant-producing strain

Freshly prepared Bhatabaru was used for the isolation and screening of LAB. Only oxidase-negative and catalase-negative strains were selected, as these two biochemical characteristics are primary features of LAB. Out of 16 isolates, 10 strains were found to be both catalase-negative and oxidase-negative and were labeled Bht1 to Bht10.

Cell-free broth was obtained by centrifuging the cultures at 10,000 rpm for 20 min after incubation in modified MRS medium (without Tween 20) at 37°C for 24 h. The resulting cell-free supernatant (CFS) from each culture was then screened for biosurfactant production using a series of assays, including hemolytic activity, drop collapse, surface activity, oil displacement, and emulsification methods, as detailed in Table 2.

**Table 2:** Screening test for different isolates for biosurfactants production

Isolate	Hemolytic activity	Drop collapse	Oil displacement method [mm]	Reduce surface tension [mN/m]	Emulsification activity
Bht-1	α	++	11.2 ± 0.3	59.33 ± 0.13	23.45 ± 0.03
Bht-2	β	+++	40.3 ± 0.6	38.14 ± 0.51	65.54 ± 0.12
Bht-3	γ	–	–	54.18 ± 0.08	33.23 ± 0.13
Bht-4	γ	–	–	–	23.56 ± 0.16
Bht-5	γ	–	-	–	17.67 ± 0.07
Bht-6	α	++	13.4 ± 0.5	69.14 ± 0.13	18.24 ± 0.03
Bht-7	γ	–	–	–	12.34 ± 0.08
Bht-8	γ	+	10.6 ± 0.2	65.04 ± 0.50	32.56 ± 0.12
Bht-9	β	+++	37.1 ± 0.8	43.03 ± 0.23	57.43 ± 0.15
Bht-10	α	++	32.5 ± 0.4	47.23 ± 0.11	52.32 ± 0.03

– = Negative, +++ = complete collapse within 1 min, ++ = collapse within 2 mins, + = collapse after 2 mins

Values are mean ± standard error of means

Among the 10 isolates screened, five strains were confirmed to be biosurfactant producers based on consistent positive results across all five screening assays.

### Hemolytic activity

Ten isolates were tested for hemolytic activity, indicated by the formation of a clear zone around the colonies. Strains Bht-2 and Bht-9 exhibited beta-hemolytic activity (complete destruction of RBCs), while Bht-1, Bht-2, Bht-6, and Bht-10 exhibited alpha-hemolytic activity (partial destruction of RBCs). In contrast, strains Bht-3, Bht-4, Bht-5, Bht-7, and Bht-8 exhibited gammahemolytic activity (no destruction of RBCs).

According to a study by Puphan et al. (2015), many biosurfactant-producing microorganisms exhibit hemolytic activity. However, some strains are capable of synthesizing biosurfactants without displaying any hemolytic activity (Rodrigues et al. 2006). Therefore, this method alone is not considered reliable for screening biosurfactant-producing strains, as numerous lytic bacteria or enzymes may lyse RBCs, producing false positives or negatives (Walter et al. 2013). This assay should be used in combination with other surface activity-based screening techniques.

**Figure 1:**
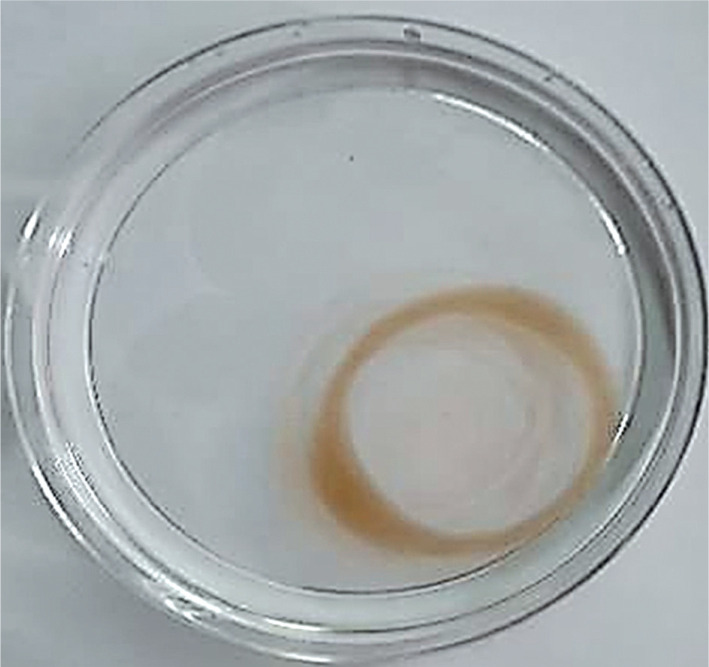
Oil displacement by supernatant of the Bht-2 strain

Oil displacement by the cell-free broth of strain Bht-2 (Figure 1) indicates the presence of biosurfactants in the supernatant. Among the ten isolates, six strains (Bht-1, Bht-2, Bht-6, Bht-8, Bht-9, and Bht-10) exhibited oil displacement, while Bht-3, Bht-4, Bht-5, and Bht-7 did not. The maximum displacement was observed in the supernatant of Bht-2 (40.3 ± 0.6 mm), followed by Bht-9 (37.1 ± 0.8 mm). The amount of biosurfactant in the culture broth directly correlates with the oil displacement area in this assay (Morikawa et al. 2000).

Biosurfactants reduce surface (liquid–air) and interfacial (liquid–liquid) tension in the cell-free supernatant, causing oil to be displaced at the interface of the two immiscible fluids (oil and water). As repulsive forces between the immiscible phases decrease, they mix and interact more readily. The oil is pushed from the surface of the water, resulting in the formation of a clear zone (Md et al. 2019).

Our results are consistent with findings by Ghasemi et al. (2019), who also screened LAB for biosurfactant production. The diameter of the clear zone on the oil surface is directly related to surfactant activity (Thaniyavarn et al. 2003; Nayarisseri et al. 2018). According to Morikawa et al. (2000), the oil spreading technique is more efficient than the drop collapse method.

**Figure 2:**
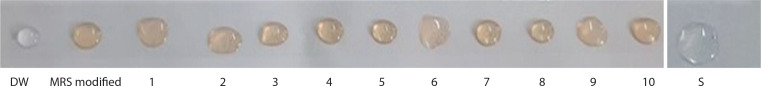
Drop collapse test is shown by different isolates of supernatant. DW – distilled water control, MRS modified – Man-Rogosa-Sharpe agar, S – positive control (SDS)

Figure 2 represents the drop collapse assay, in which droplets 1–10 correspond to different isolates. DW represents the distilled water control, and S denotes the SDS (positive control) droplet. In this assay, variations in the droplet diameter on thin parafilm indicate biosurfactant activity. The drop collapse method is based on the destabilization of liquid droplets.

The supernatants from strains Bht-3, Bht-4, Bht-5, and Bht-7 did not cause droplet collapse, whereas the droplets from strains Bht-2 and Bht-9 collapsed immediately within 1 minute. Droplet flattening occurred within 2 min for strains Bht-1, Bht-6, and Bht-10, while Bht-8 showed flattening after 2 min.

In the absence of surfactants, polar water molecules adhere to the hydrophobic surface, and the droplet remains stable. However, when surfactants are present, the interfacial tension between the liquid droplet and the hydrophobic surface is reduced, causing the droplet to spread or collapse. Drop stability is thus influenced by surfactant concentration and is directly related to surface and interfacial tension.

These findings are consistent with those of Cornea et al. (2016), who used the drop collapse method to identify biosurfactant-producing microbes (Ghazi et al. 2023). Many researchers utilized the drop collapse test for screening biosurfactant-producing isolates in their investigations (Tugrul and Cansunar 2005). It can still be employed even when biosurfactants quantity is low.

Emulsification activity also indicates the presence of biosurfactants, as it reflects the ability to mix two or more immiscible liquids into a semi-stable emulsion. Maximum emulsification activity was observed in isolate Bht-2 (65.54 ± 0.12), followed by Bht-9 (57.43 ± 0.15) and Bht-10 (52.32 ± 0.03). Isolates Bht-1, Bht-3, Bht-7, and Bht-8 exhibited moderate emulsification activity, as shown in Table 2. In many studies, this method has been utilized for the selection of the biosurfactant-producing microorganisms (Hisham et al. 2019). Some studies suggest a direct correlation between surface activity and emulsification capacity (Nayarisseri et al. 2018), though others report that stable emulsions are not always linked to surface tension-lowering activity (Noha et al. 2004).

Surface activity is a critical parameter in screening biosurfactant-producing strains. Strain Bht-2 showed the highest surface tension reduction, from 72.2 mN/m to 38.14 ± 0.51, followed by Bht-9 (43.03 ± 0.23) and Bht-10 (47.23 ± 0.11). A surface tension value below 45 dynes/cm is generally considered a positive indicator in the drop collapse test, which aligns with the findings of this study (Ramos et al. 2010). Strong biosurfactants can reduce the surface tension of distilled water from 72.0 to approximately 35.0 mN/m. The biosurfactant produced by strain Bht-2 significantly reduced surface tension to 38.14 ± 0.51 (Sharma et al. 2015). Similarly, *Lactococcus lactis* 53 and *Streptococcus thermophilus* A have been reported to reduce surface tension to approximately 36–37.0 mN/m (Sharma et al. 2015).

Based on all the screening tests for biosurfactant production, five out of ten isolates – Bht-1, Bht-2, Bht-6, Bht-9, and Bht-10 – were confirmed as biosurfactant producers.

### Biomass and biosurfactants determination

Biomass and biosurfactant concentrations were determined for the selected isolates (Bht-1, Bht-2, Bht-6, Bht-9, and Bht-10).

As depicted in Table 3, among five isolates (Bht-1, Bht-2, Bht-6, Bht-9, and Bht-10), the biomass and biosurfactants yield of the Bht-2 strain was comparatively higher than that of the other isolates. The biomass of Bht-2 was determined as 0.675 ± 0.03, and biosurfactant yield was estimated as 0.21 ± 0.05, respectively. According to Ghasemi et al. (2020) biosurfactants yielded from LAB *Pediococcus dextrinicus* were reported to be 0.7g/l, and biosurfactant yield for *Lactobacillus lactis* was 0.1–4.6 g/l as reported by Souza et al. (2017). The biosurfactant yield of four isolated strains was higher compared to the reported studies. Based on the high yield for biosurfactants production, the Bht-2 strain was further selected and characterized. The difference in the yield of biosurfactants was due to the different nature of biosurfactant-producing strains. The yield was also affected by many factors, such as variations in culture conditions like temperature, pH, different media composition, etc.

**Table 3:** Biomass and biosurfactants determination of different isolates

Isolates	Biomass determination [g/150 ml]	Biosurfactant determination [g/150 ml]
Bht-1	0.322 ± 0.03	0.045 ± 0.06
Bht-2	0.675 ± 0.03	0.21 ± 0.05
Bht-6	0.48 ± 0.04	0.105 ± 0.01
Bht-9	0.435 ± 0.02	0.09 ± 0.02
Bht-10	0.555 ± 0.03	0.135 ± 0.03

Values are mean ± standard error of means

Among all biosurfactant-producing isolates, strain Bht-2 exhibited the highest biosurfactant yield and the greatest surface tension reduction. Therefore, it was selected for further morphological, biochemical, and genetic identification.

Morphological analysis of the selected strain, as shown in Table 4, revealed that Bht-2 was Gram-positive, coccus-shaped, off-white in color, and nonendospore forming. Table 4 also presents the biochemical characterization results. The strain tested negative for catalase, oxidase, MR, indole, and H_2_S production, while it tested positive for the VP reaction and nitrate reduction.

**Table 4:** Characterization of strain Bht-2

Morphological characterization									
Colour						Off white			
Motility						–			
Cell shape (negative staining)						Coccus			
Gram staining						+			
Endospore						–			
**Biochemical characterization**									
Catalase						–			
Oxidase						–			
Methyl red						–			
VP reaction						+			
Indole						–			
Citrate						–			
Nitrate reduction						+			
H_2_S						–			
**Carbohydrate utilization test**									
Lactose	–	Trehalose	+	Glycerol	+	a-methyl-D-glucoside	–	Esculin hydrolysis	+
Xylose	–	Melibiose	–	Dulcitol	+	Rhamnose	+	D-Arabinose	–
Maltose	–	Sucrose	+	Inositol	+	Cellobiose	+	Citrate utilization	–
Fructose	+	L-arabinose	–	Sorbitol	+	Melezitose	+	Sorbose	+
Dextrose	+	Mannose	–	Mannitol	+	a-methyl-D-mannoside	+	Erythritol	–
Galactose	+	Insulin	+	Adonitol	–	Xylitol	–	Salicin	+
Raffinose	+	Sodium gluconate	+	Arabitol	–	ONPG	–	Erthritol	–

### Carbon utilization test

As depicted in Table 4 selected strain Bht-2 utilized a total of 19 carbon sources i.e., fructose, dextrose, galactose, raffinose, trehalose, inulin, sodium gluconate, salicin, dulcitol, inositol, sorbitol, mannitol, rhamnose, cellobiose, melezitose, a-methyl-mannoside, esculin hydrolysis and sorbose out of 35 carbon sources.

Morphological analysis of strain Bht-2 (also in Table 4) confirmed that the isolate was Gram-positive, coccus-shaped, off-white in color, and lacked endospore formation. Biochemical testing showed the strain was negative for catalase, oxidase, MR, indole, and H_2_S production, and positive for VP reaction and nitrate reduction.

These results are consistent with findings reported by Lebreton et al. (2014) and Růžičková et al. (2020). The majority of the results pattern of carbon utilization of the selected strain was reported by the two studies by Hagedorn et al. (2003), Ramsey (2014) for carbon sources, but for lactose, raffinose, inositol, and sorbose result differs. The difference in the result of the four carbon sources might be due to the isolation of the strain from different sources.

### Genotypic and phylogenetic identification

For genotypic and phylogenetic analysis, DNA was isolated from the Bht-2 strain and subjected to amplification.

**Figure 3:**
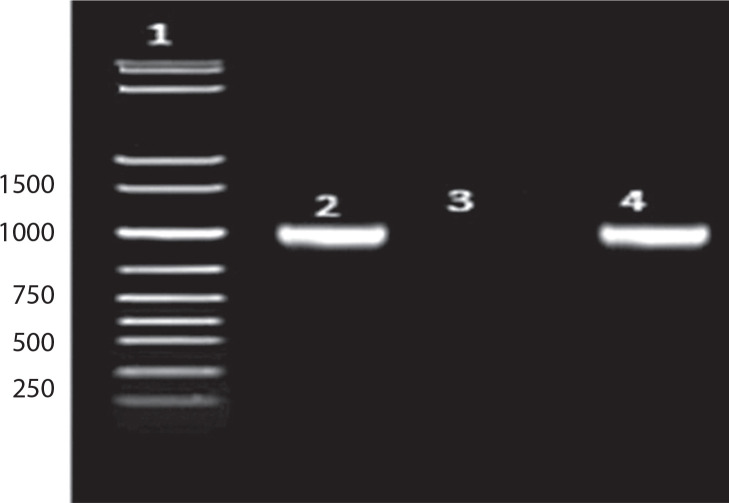
PCR product of 16S rRNA on 2% agarose gel (description of the lanes 1-4 in the text)

As shown in Figure 3: Lane 1: 1 kb DNA ladder; Lane 2: 16S amplicon (positive control); Lane 3: Negative control; Lane 4: Sample (Bht-2).

### Phylogenetic analysis of Enterococcus faecalis based on 16S rRNA sequence

A preliminary comparison of the 16S rRNA sequence of strain Bht-2 with sequences available in GenBank revealed that the isolate belongs to the *Enterococcus* genus. The phylogenetic analysis of the 16S rRNA sequence also validated the genus *Enterococcus* sp., which proved to be the closest match in the BLAST with 99.85 percent homology with *E. faecalis.* The 16S rRNA sequence of Bht-2 was submitted to GenBank (NCBI, USA), and the assigned accession number is OM843218.

Biosurfactants derived from *E. faecalis* hold considerable potential for various applications. However, safety remains a critical consideration. While some *E. faecalis* strains serve as probiotics, others may harbor antibiotic resistance genes and virulence factors, posing a risk of opportunistic infections, especially in immunocompromised individuals.

**Figure 4:**
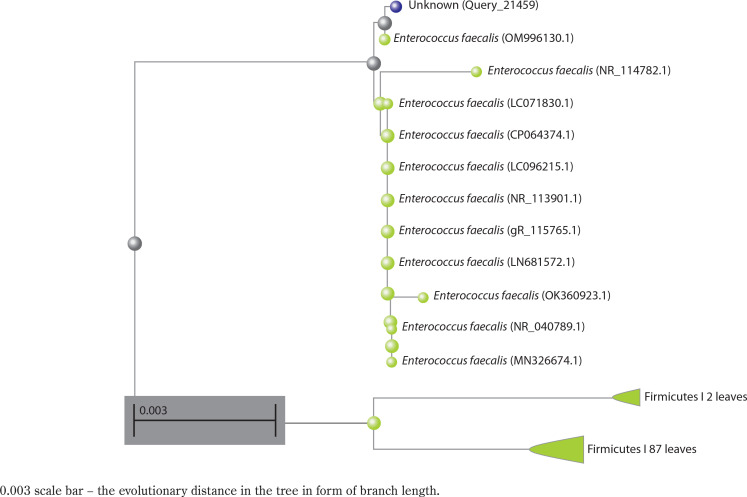
Phylogenetic analysis of selected strain Bht-2

Research on biosurfactant production by *Enterococcus* strains remains limited. Future studies should focus on optimizing production, purification, and application of biosurfactants, while also conducting thorough genomic, toxicological, and functional evaluations to ensure safety. Strain-specific safety profiling will be essential to mitigate potential health risks and enable the safe use of *E. faecalis*-derived biosurfactants in therapeutic and industrial contexts.

## Conclusions

Fermented foods are rich sources of LAB, which enhance flavor, texture, and nutritional value while producing bioactive metabolites such as biosurfactants and bacteriocins. In this study, a biosurfactant-producing LAB strain was isolated and characterized from Bhatabaru, a traditional Indian fermented food. Among the isolates, strain Bht-2 exhibited the highest biosurfactant activity and was identified as *Enterococcus faecalis* through morphological, biochemical, and genetic analyses. This strain produced 0.675 ± 0.03 g of biomass and 0.21 ± 0.05 g of biosurfactant.

Further investigations are needed to elucidate the chemical structure of the biosurfactant and assess its antimicrobial and antiadhesive properties against biofilmforming pathogens. Comprehensive safety assessments will also be essential to identify and mitigate potential health risks, ensuring its safe application. These findings highlight the potential of strain Bht-2 for broader use in therapeutic, industrial, and biotechnological applications.
